# Supplementary material for: Sediment Bacteria and Phosphorus Fraction Response, Notably to Titanium Dioxide Nanoparticle Exposure
Source: Microorganisms. 2022 Aug 13;10(8):1643. doi: 10.3390/microorganisms10081643 (PMC9412993; doi:10.3390/microorganisms10081643)
Supplement: Supplementary file 1 [file microorganisms-10-01643-s001.zip › microorganisms-1854833-supplementary.pdf]

Supplementary materials for:

Sediment Bacteria and Phosphorus Fraction Response, Notably to Titanium Dioxide Nanoparticle Exposure

Sixuan Piao and Donglan He *

Hubei Provincial Engineering and Technology Research Center for Resources and Utilization of Microbiology, College of Life Science, South-Central Minzu University, Wuhan 430074, China
* Correspondence: hdl@mail.scuec.edu.cn

Supplementary material for methods:

Experiment 1: Amplification for phosphorus-cycling-related genes

Primer gcdF (5'–CGG CGT CAT CCG GGS NTN YRA YRT–3') and primer gcdR (5'–GGG CAT GTC CAT GTC CCA NAD RTC RTG–3') were applied to amplify *gcd* gene (Cleton-Jansen et al. 1990); Primer ALPS-F730 (5'–CAG TGG GAC GAC CAC GAG GT–3') and primer ALPS-R1101 (5'–GAG GCC GAT CGG CAT GTC G–3') were used to amplify *phoD* gene (Sakurai et al. 2008); Primer pstSF (5'–TCT ACC TGG GGA AGA TCA CAA ART GGR A–3') and primer pstSR (5'–TGC CGA CGG GCC ANT YNW C–3') were selected to amplify *pstS* gene (Hsieh and Wanner 2010).

Standard curves were generated using a serial dilution of a known amount of linearized recombinant plasmid containing specific gene fragment. Quantitation was performed on three technical replicates with an ABI VIIA 7 Cycle Real-time PCR System (Applied Biosystems, Foster City, CA, USA) in a 10 μL reaction system, and was conducted at 95 °C for 5 min, followed by 40 cycles of 95 °C for 15 s and 55 °C for 1 min. The amplification efficiencies for these P-cycling-related genes were as follows: *gcd*: 97%, *phoD*: 105%, and *pstS*: 99%. The abundances of these genes in all samples were expressed as copies per gram of freeze-dried sediment.

Reference

Cleton-Jansen AM, Goosen N, Fayet O, van de Putte P (1990) Cloning, mapping, and sequencing of the gene encoding *Escherichia coli* quinoprotein glucose dehydrogenase. J Bacteriol 172:6308–6315.

Hsieh YJ, Wanner BL (2010) Global regulation by the seven-component pi signaling system. Curr Opin Microbiol 13:198–203.

Sakurai M, Wasaki J, Tomizawa Y, Shinano T, Osaki M (2008) Analysis of bacterial communities on alkaline phosphatase genes in soil supplied with organic matter. Soil Sci Plant Nutr 54:62–71.

Table S1 Content of phosphorus fractions in both water and sediment

Sample	SRP[c] (mg/L)	TSP (mg/L)	WTP (mg/L)	TP (mg/g)	Olsen P (mg/g)	IP (mg/g)	OP (mg/g)	AP (mg/g)	NAIP (mg/g)	Pmb (mg/g)
A1T1[a]-R1[b]	0.112	0.125	0.632	1.13	0.21	0.98	0.15	0.83	0.3	0.367
A1T1-R2	0.135	0.122	0.553	1.24	0.23	1.08	0.16	0.75	0.49	0.387
A1T1-R3	0.123	0.123	0.612	1.26	0.22	1.19	0.07	0.72	0.54	0.413
A1T2-R1	0.156	0.137	0.586	1.27	0.24	1.09	0.18	0.57	0.7	0.367
A1T2-R2	0.147	0.158	0.612	1.23	0.23	1.12	0.11	0.95	0.28	0.349
A1T2-R3	0.152	0.123	0.544	1.34	0.24	1.07	0.27	0.74	0.6	0.363
A1T3-R1	0.143	0.156	0.558	1.24	0.25	0.95	0.29	0.65	0.59	0.358
A1T3-R2	0.172	0.199	0.562	1.1	0.27	0.93	0.17	0.65	0.45	0.384
A1T3-R3	0.164	0.126	0.601	1.11	0.29	0.84	0.27	0.75	0.36	0.325
A2T1-R1	0.125	0.127	0.571	1.28	0.22	1.19	0.09	0.67	0.61	0.354
A2T1-R2	0.128	0.129	0.498	1.4	0.26	1.28	0.12	0.67	0.73	0.325
A2T1-R3	0.135	0.108	0.488	1.34	0.24	0.94	0.4	0.56	0.78	0.373
A2T2-R1	0.167	0.159	0.497	1.26	0.31	1.02	0.24	0.76	0.5	0.326
A2T2-R2	0.158	0.178	0.502	1.36	0.22	1.19	0.17	0.68	0.68	0.368
A2T2-R3	0.163	0.186	0.432	1.31	0.25	1.14	0.17	0.67	0.64	0.356
A2T3-R1	0.185	0.187	0.601	1.21	0.33	0.99	0.22	0.74	0.47	0.313
A2T3-R2	0.178	0.208	0.424	1.51	0.26	1.23	0.28	0.83	0.68	0.287
A2T3-R3	0.192	0.171	0.502	1.21	0.28	1.01	0.2	0.67	0.54	0.302
A3T1-R1	0.125	0.126	0.441	1.51	0.26	1.21	0.3	0.76	0.75	0.289
A3T1-R2	0.132	0.124	0.501	1.59	0.27	1.31	0.28	0.83	0.76	0.326
A3T1-R3	0.135	0.138	0.436	1.66	0.32	1.41	0.25	0.72	0.94	0.332
A3T2-R1	0.178	0.198	0.443	1.54	0.35	1.28	0.26	0.73	0.81	0.314
A3T2-R2	0.182	0.204	0.435	1.61	0.25	1.37	0.24	0.56	1.05	0.278
A3T2-R3	0.191	0.203	0.467	1.35	0.28	1.25	0.1	0.78	0.57	0.254
A3T3-R1	0.197	0.181	0.474	1.48	0.19	1.27	0.21	0.57	0.91	0.258
A3T3-R2	0.188	0.195	0.437	1.55	0.35	1.34	0.21	0.91	0.64	0.302

A3T3-R3	0.193	0.201	0.485	1.57	0.26	1.53	0.04	0.87	0.7	0.264
A4T1-R1	0.165	0.168	0.434	1.46	0.32	1.11	0.35	0.79	0.67	0.235
A4T1-R2	0.163	0.179	0.429	1.39	0.34	1.38	0.01	0.87	0.52	0.252
A4T1-R3	0.172	0.167	0.431	1.58	0.33	1.35	0.23	1.07	0.51	0.247
A4T2-R1	0.179	0.204	0.456	1.34	0.32	1.02	0.32	1.02	0.32	0.301
A4T2-R2	0.192	0.189	0.421	1.51	0.34	1.37	0.14	0.81	0.7	0.213
A4T2-R3	0.199	0.202	0.398	1.57	0.38	1.22	0.35	1.03	0.54	0.233
A4T3-R1	0.205	0.221	0.401	1.63	0.43	1.51	0.12	1.08	0.55	0.241
A4T3-R2	0.206	0.232	0.421	1.51	0.36	1.21	0.3	1.16	0.35	0.202
A4T3-R3	0.189	0.199	0.425	1.82	0.39	1.35	0.47	0.92	0.9	0.258
A5T1-R1	0.211	0.225	0.398	1.88	0.37	1.11	0.77	0.84	1.04	0.197
A5T1-R2	0.175	0.168	0.387	1.98	0.39	1.37	0.61	1.23	0.75	0.202
A5T1-R3	0.189	0.212	0.329	1.63	0.41	1.29	0.34	1.26	0.37	0.219
A5T2-R1	0.221	0.205	0.345	1.82	0.43	1.33	0.49	1.41	0.41	0.252
A5T2-R2	0.235	0.254	0.378	1.61	0.45	1.28	0.33	1.32	0.29	0.237
A5T2-R3	0.267	0.302	0.425	1.71	0.53	1.06	0.65	1.53	0.18	0.211
A5T3-R1	0.253	0.334	0.414	1.84	0.37	1.28	0.56	1.28	0.56	0.187
A5T3-R2	0.198	0.243	0.397	1.78	0.62	1.43	0.35	1.22	0.56	0.243
A5T3-R3	0.242	0.329	0.357	1.86	0.53	1.21	0.65	1.55	0.31	0.205

[a], A and T represent TiO2 NPs amount levels (Amount1, Amount2, Amount3, Amount4, Amount5) and incubation time (T1 for day 1, T2 for day 10, and T3 for day 30).

[b], R1, R2, and R3 denote three replicates.

[c], Abbreviations of SRP, TSP, WTP, TP, IP, OP, AP, NAIP, and Pmb denote soluble reactive phosphorus, total soluble phosphorus, water total phosphorus, total phosphorus, inorganic phosphorus, organic phosphorus, non-apatite inorganic phosphorus, and apatite phosphorus.

Table S2 Topological parameters of co-occurrence networks for bacteria in sediments with different titanium dioxide nanoparticles addition (i.e., Amount1, Amount2, Amount3, Amount4, Amount5, and Amount6).

Property	Amount1	Amount2	Amount3	Amount4	Amount5	All
Node	32	37	30	31	30	40
Edge	92	95	135	68	148	305
Positive edge /Negative edge	1.88	0.94	1.08	3	0.97	1.68
Module	5	5	5	5	5	3
Average degree	5.25	4.87	7	3.81	7.73	9.85
Diameter	7	7	5	8	8	5
Graph density	0.169	0.135	0.241	0.127	0.267	0.253
Average clustering coefficient	0.509	0.432	0.626	0.528	0.598	0.526
Average path length	2.650	2.846	2.135	3.327	2.420	2.096

Table S3 Pearson Correlation between phosphorus components and relative abundances of top 10 phyla.

	SRP	TSP	WTP	TP	Olsen P	IP	OP	AP	NAIP	Pmb
Actinobacteria	0.222	0.194	-0.034	-0.120	0.130	0.037	-0.196	0.131	-0.301*	-0.167
Proteobacteria	-0.485***	-0.454**	-0.018	0.069	-0.199	0.064	0.030	-0.107	0.213	0.110
Acidobacteria	0.056	0.083	0.382**	-0.365*	-0.160	-0.307*	-0.191	-0.221	-0.122	0.383*
Chloroflexi	0.249	0.224	0.254	-0.345*	-0.053	-0.273	-0.199	-0.123	-0.255	0.204
Firmicutes	0.176	0.158	-0.445**	0.587***	0.285	0.342*	0.455**	0.293	0.275	-0.382**
Bacteroidetes	-0.483***	-0.436**	0.109	-0.019	-0.198	-0.071	0.043	-0.154	0.176	0.166
Gemmatimonadetes	0.018	-0.014	0.031	-0.212	-0.020	-0.008	-0.276	-0.048	-0.175	-0.094
Rokubacteria	0.127	0.141	0.307*	-0.299*	-0.154	-0.275	-0.135	-0.189	-0.090	0.295*
Patescibacteria	-0.124	-0.133	-0.066	0.006	0.006	0.098	-0.086	0.013	-0.010	-0.145
Planctomycetes	-0.184	-0.193	0.350*	-0.225	-0.110	-0.214	-0.095	0.037	-0.298*	0.221

Note: Asterisks denote significance (*, $p < 0.05$; **, $p < 0.01$; ***, $p < 0.001$). Abbreviations: SRP, soluble reactive phosphorus; TSP, total soluble phosphorus; WTP, water total phosphorus; TP, total phosphorus; IP, inorganic phosphorus; AP, total apatite inorganic P; NAIP, non-apatite inorganic P; Pmb, microbial biomass phosphorus.

Table S4 Pearson correlations between phosphorus components and functions at KEGG pathway level 2.

	SRP	TSP	WTP	TP	Olsen P	IP	OP	AP	NAIP	Pmb
BOOSM	0.020	0.040	0.135	-0.192	0.011	-0.024	-0.233	-0.046	-0.154	-0.072
MOOAA	0.223	0.220	-0.074	0.125	0.109	0.052	0.118	0.147	-0.049	0.028
MOTAP	-0.558***	-0.527***	0.251	-0.320*	-0.356*	-0.093	-0.339*	-0.230	-0.060	0.262
EM	0.473***	0.463***	-0.088	0.156	0.217	0.045	0.166	0.148	-0.016	-0.051
LM	-0.460***	-0.424**	0.441**	-0.529***	-0.477***	-0.211	-0.504***	-0.342*	-0.149	0.485***
XBAM	-0.159	-0.197	0.396**	-0.558***	-0.341*	-0.354*	-0.405**	-0.326*	-0.202	0.397**
GBAM	-0.303*	-0.260	-0.041	0.005	-0.052	0.204	-0.190	0.008	-0.005	-0.119
CM	-0.276	-0.266	-0.114	0.183	-0.045	0.075	0.172	0.013	0.186	0.032
MT	-0.065	-0.075	-0.301*	0.402**	0.111	0.153	0.390**	0.143	0.262	-0.151
Translation	0.565***	0.571***	-0.347*	0.444**	0.442**	0.307*	0.299*	0.324*	0.079	-0.432**
Transcription	0.567***	0.564***	-0.311*	0.384**	0.431**	0.224	0.298*	0.302*	0.039	-0.409**
RAR	0.530***	0.536***	-0.326*	0.416**	0.439**	0.278	0.288	0.322*	0.049	-0.445**

Asterisks denote significance (*, $p < 0.05$; **, $p < 0.01$; ***, $p < 0.001$). Abbreviations for functions: BOOSM, Biosynthesis of other secondary metabolites; MOOAA, Metabolism of other amino acids; MOTAP, Metabolism of terpenoids and polyketides; EM, Energy metabolism; LM, Lipid metabolism; XBAM, Xenobiotics biodegradation and metabolism; GBAM, Glycan biosynthesis and metabolism; CM, Cell motility; Membrane transport, MT; RAM, Replication and repair. SRP, soluble reactive phosphorus; TSP, total soluble phosphorus; WTP, water total phosphorus; TP, total phosphorus; IP, inorganic phosphorus; AP, total apatite inorganic P; NAIP, non-apatite inorganic P; Pmb, microbial biomass phosphorus.

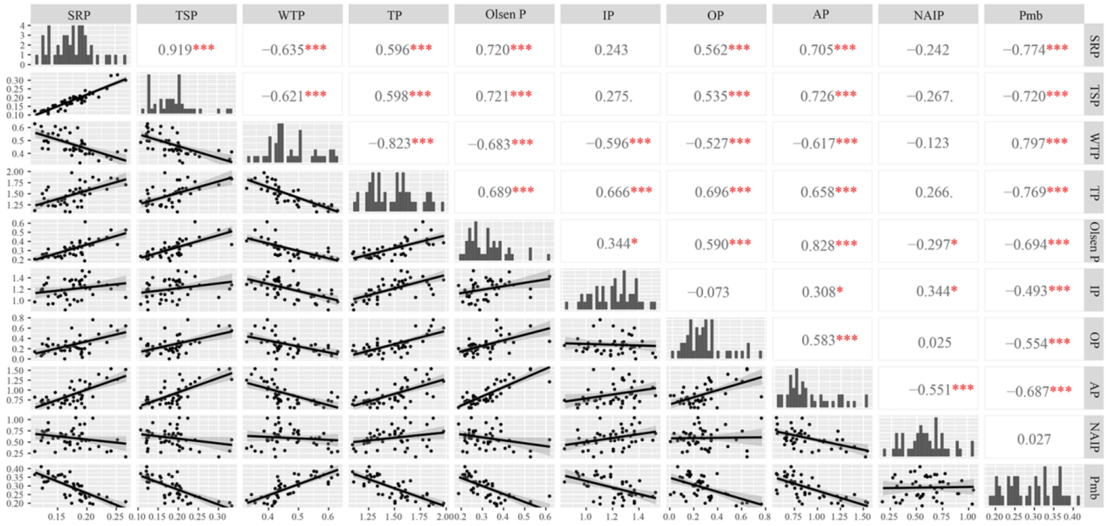

Figure S1. Pearson correlations among phosphorus fractions. Asterisks represent significance (*, $p < 0.05$; ***, $p < 0.001$). Abbreviations: SRP, soluble reactive phosphorus; TSP, total soluble phosphorus; WTP, water total phosphorus; TP, total phosphorus; IP, inorganic phosphorus; AP, total apatite inorganic P; NAIP, non-apatite inorganic P; Pmb, microbial biomass phosphorus.

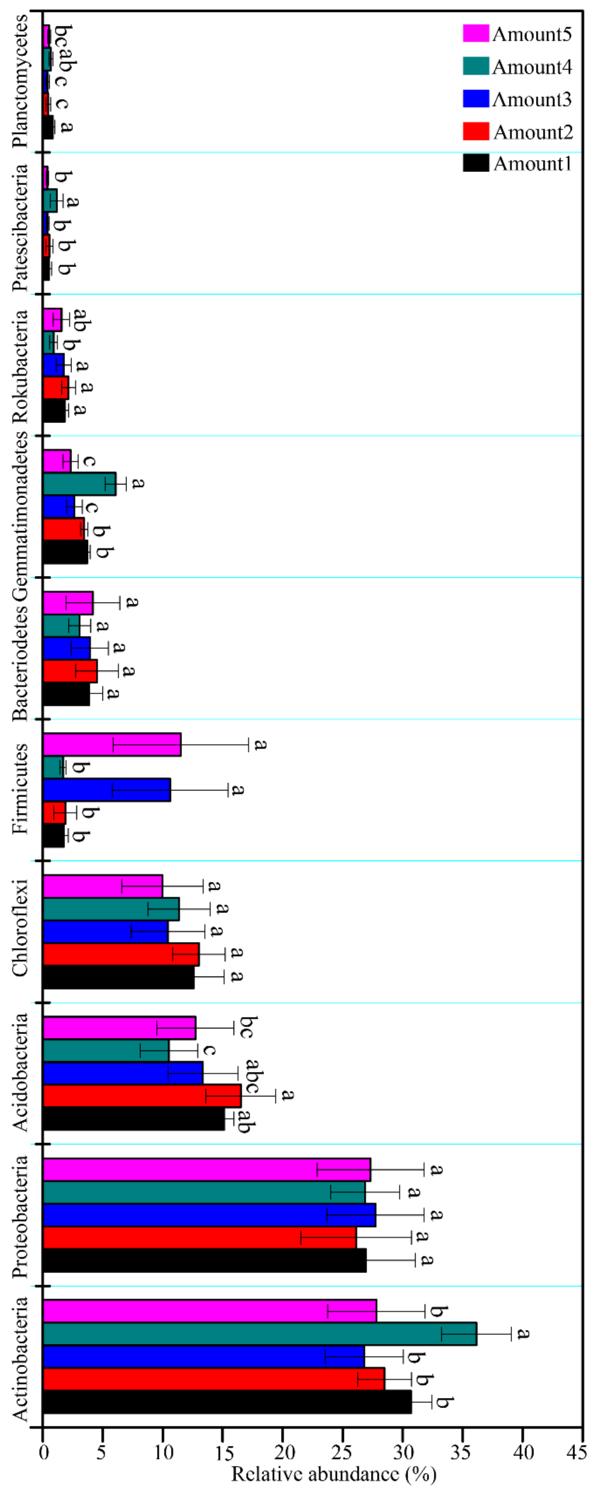

Figure S2. Differences in relative abundances of top 10 phyla among five groups with different TiO_2 NPs addition amounts. Different letters above column denote significant levels ($p < 0.05$).
